# Supplementary material for: Newtic1 Is a Component of Globular Structures That Accumulate along the Marginal Band of Erythrocytes in the Limb Blastema of Adult Newt, Cynops pyrrhogaster
Source: Biomedicines. 2022 Nov 1;10(11):2772. doi: 10.3390/biomedicines10112772 (PMC9687212; doi:10.3390/biomedicines10112772)
Supplement: Supplementary file 1 [file biomedicines-10-02772-s001.zip › biomedicines-1988402-supplementary.pdf]

Supplementary Information

for

Newtic1 is a component of globular structures that accumulate along the marginal band of erythrocytes in the limb blastema of adult newt, *Cynops pyrrhogaster*

Xutong Chen, Ryo Ando, Roman Martin Casco-Robles, Martin Miguel Casco-Robles, Fumiaki Maruo, Shuichi Obata & Chikafumi Chiba

SO: obatashu@kitasato-u.ac.jp; CC: chichiba@biol.tsukuba.ac.jp

Supplementary Table S1. Antibodies for immunostaining.

Antibody	Dilution (IgG conc.)	Provider
**Primary**		
Rabbit anti-Newtic1 polyclonal antibody	1:200 (2.1 µg/mL)	Custom made; Merck Sigma-Aldrich, Tokyo, Japan
Rabbit anti-TGFB1 polyclonal antibody	1:500 (2.0 µg/mL)	LS-B14345; LifeSpan BioSciences, Inc., Seattle, WA, USA
Rabbit anti-RFP polyclonal antibody	1:500 (2.0 µg/mL)	600-401-379; Rockland Immunochemicals, city, PA, USA
Rabbit anti-BMP2 polyclonal antibody	1:500 (2.0 µg/mL)	LS-B13128; LifeSpan BioSciences, InC., Seattle, WA, USA
Rabbit anti-alpha tubulin polyclonal antibody	Figure 2<br>1:100-1:1000<br>(0.2-1.8 µg/mL)<br><br>Figure 5<br>1:500<br>(0.4 µg/mL)	Ab15246; abcam, Cambridge, UK
Mouse anti-acetylated tubulin monoclonal antibody	1:1000 (2.2 µg/mL)	T6793; Merck Sigma-Aldrich, Tokyo, Japan
**Secondary**		
Rhodamine (TRITC)-conjugated AffiniPure goat anti-rabbit IgG (H+L) polyclonal antibody	1:500 (1.5 µg/mL)	111-025-003; Jackson ImmunoResearch Laboratories, West Grove, PA, USA
Alexa Fluor 488-conjugated goat anti-rabbit IgG (H+L) polyclonal antibody	1:500 (4.0 µg/mL)	A11008; Thermo Fisher Scientific, Tokyo, Japan
Alexa Fluor 488-conjugated AffiniPure Fab Fragment goat anti-rabbit IgG (H+L) polyclonal antibody	1:500 (1.4 µg/mL)	111-025-003; Jackson ImmunoResearch Laboratories, West Grove, PA, USA
Alexa Fluor 488-conjugated goat anti-mouse IgG (H+L)	1:500 (4.0 µg/mL)	A11001; Thermo Fisher Scientific, Tokyo, Japan
Biotinylated goat anti-rabbit IgG (H+L) polyclonal antibody	1:500 (3.0 µg/mL)	BA-1000; Vector laboratories, Newark, CA, USA
Goat anti-Rabbit IgG (H&L) Ultra Small	1:50 (1.2-1.6 µg/mL)	800011; AURION Immuno Gold Reagents & Accessories, Wageningen, the Netherlands

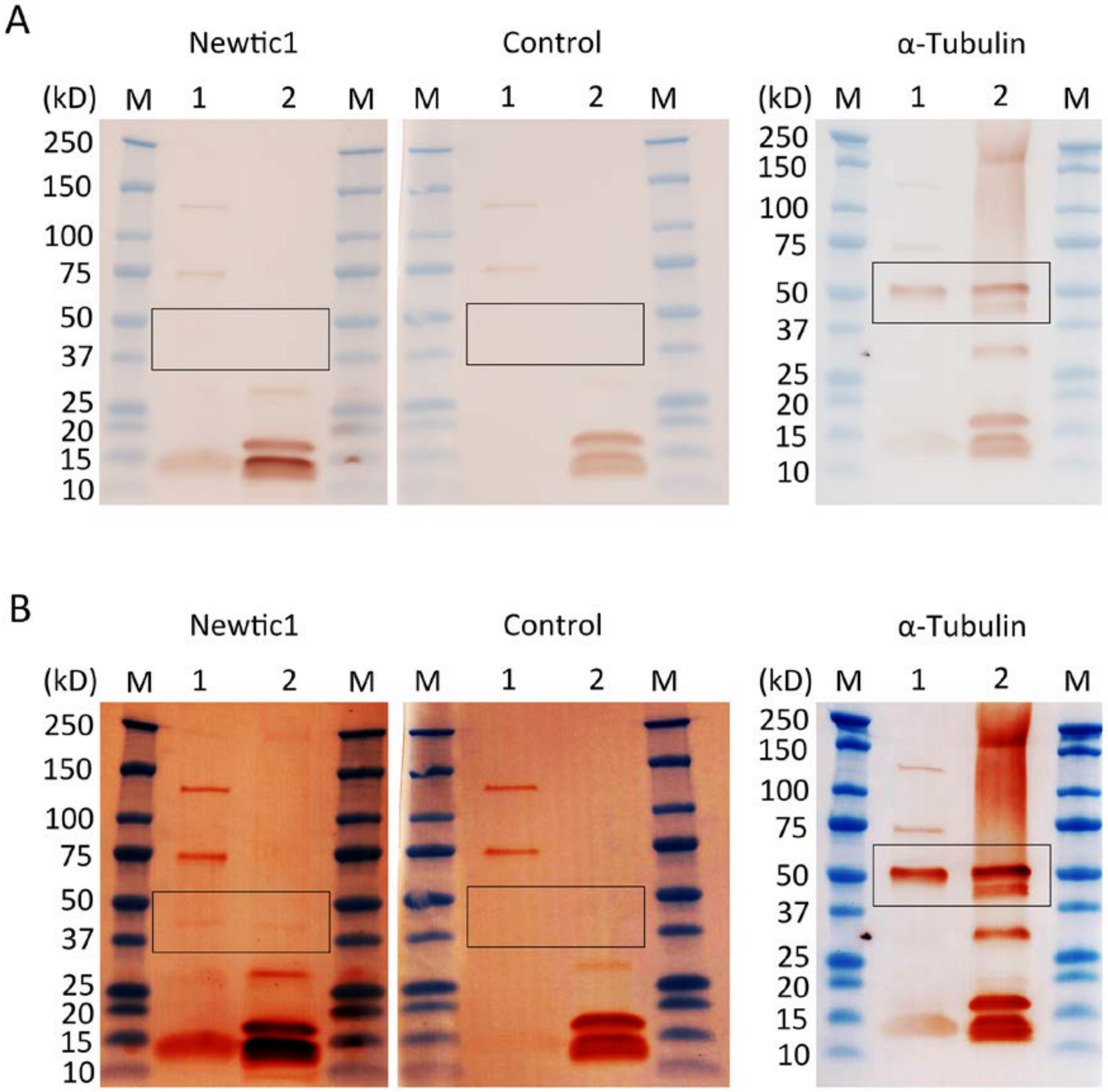

Supplementary Figure S1. Originals of Figure 5B. (**A**) Raw data. (**B**) Contrast-enhanced images of the raw data. Protein bands in the boxes in **B** were shown in Figure 5B.

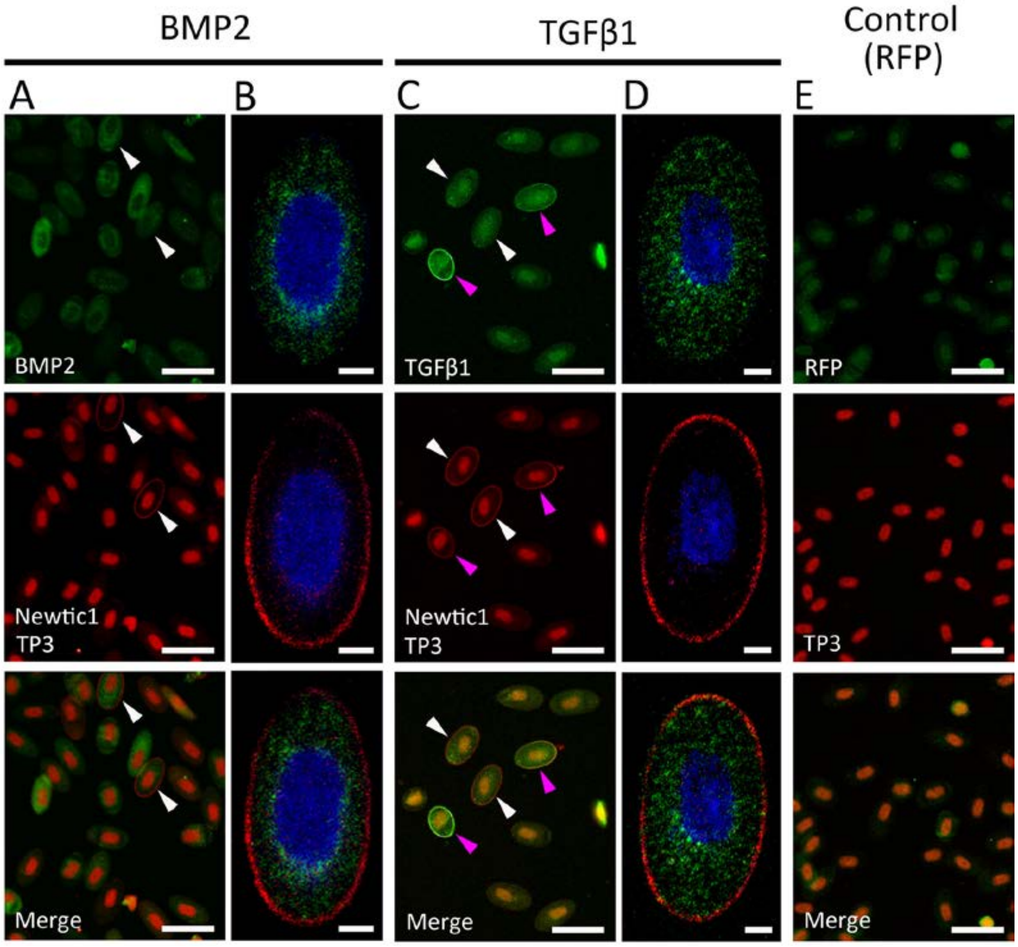

Supplementary Figure S2. Expression of BMP2 and TGFβ1 in normal circulating PcNobs. (**A, B**) A representative set of images of BMP2 and Newtic1 double stain. (**A**) Normal fluorescence microscopy images. Almost all PcNobs had BMP2 immunoreactivity (green) in the cytoplasm, albeit of varying intensity. Their immunoreactivity was intense around the nucleus and decreased toward the periphery. Arrowheads indicate Newtic1(+) PcNobs, which showed red fluorescence in a ring along the equator. In the optics here, nuclei stained with TO-PRO-3 (TP3) were detected in red. (**B**) Confocal images of a typical Newtic1(+) PcNob. There was little overlap between the immunoreactivity of Newtic1 (red) along the equator and that of BMP2 (green) in the cytoplasm. Blue: TO-PRO-3 nuclear stain. (**C, D**) A representative set of images of TGFβ1 and Newtic1 double stain. (**C**) Normal fluorescence microscopy images. Almost all PcNobs had TGFβ1 immunoreactivity (green) in the cytoplasm, albeit of varying intensity. In most cells, their immunoreactivity was intense around the nucleus and decreased toward the periphery, as observed with BMP2, but in a small number of cells, immunoreactivity was also observed along the equator. White arrowheads indicate Newtic1(+)PcNobs, whose Newtic1 immunoreactivity along the equator did not overlap with that of TGFβ1. Magenta arrowheads indicate Newtic1(+) PcNobs, in which TGFβ1 immunoreactivity was observed along their equator. Nuclear stain with TP3 is shown in red. (**D**) Confocal images of a typical Newtic1(+) PcNob without TGFβ1

immunoreactivity along the equator. Blue: TO-PRO-3 nuclear stain. (**E**) A representative set of normal fluorescence microscopy images of RFP and Newtic1 double stain. Note that all primary antibodies used here were produced by a rabbit. Therefore, staining with Newtic1 antibody was preceded by staining with the other primary antibodies. Scale bars: 100 µm (**A, C, E**); 5 µm (**B, D**).

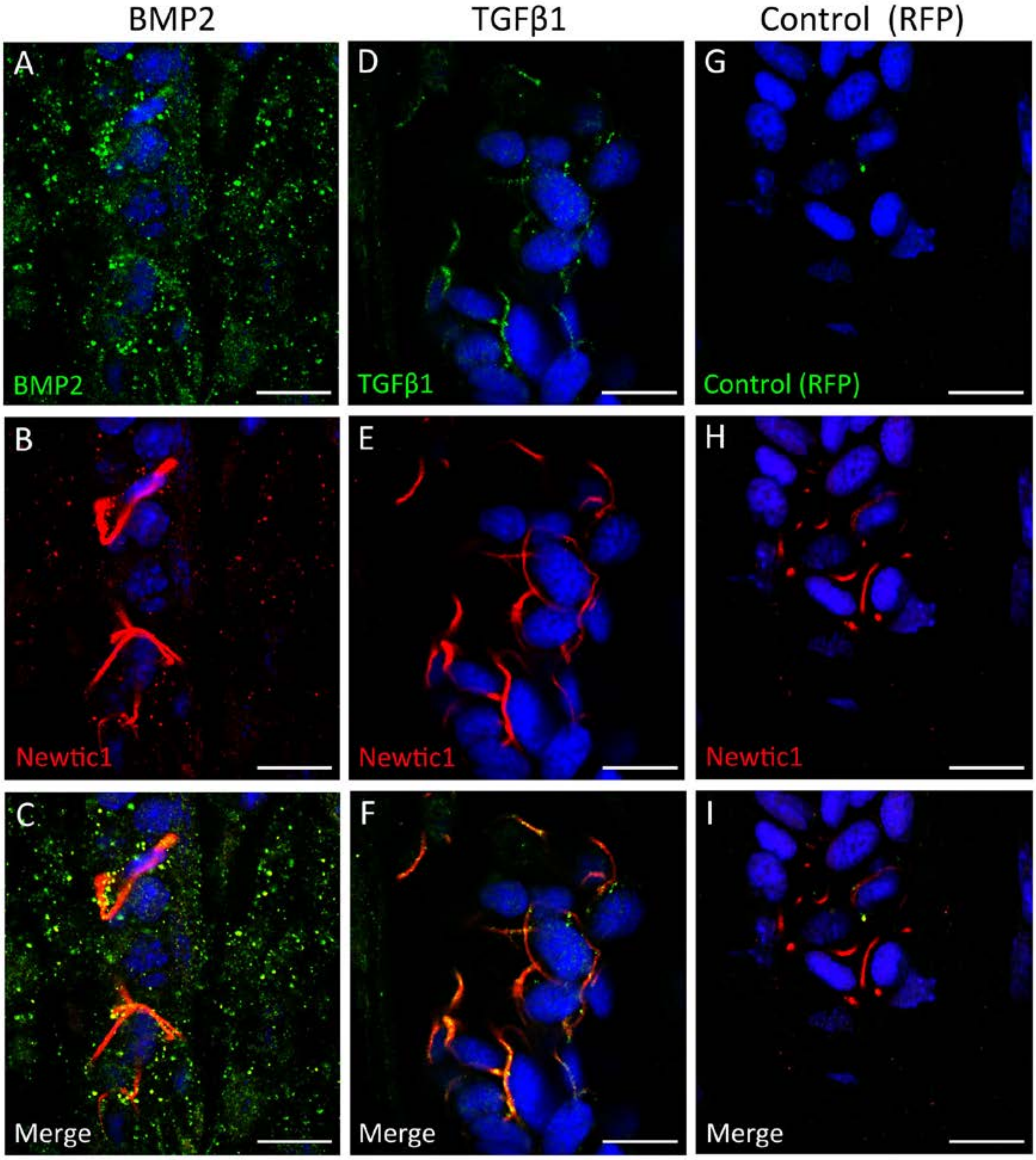

Supplementary Figure S3. Expression patterns of BMP2 and TGFβ1 in PcNobs in the limb blastema. (**A-C**) A representative set of confocal images of BMP2 and Newtic1 double stain. BMP2 immunoreactivity (green) was scattered throughout the tissue. In PcNobs, which had accumulated in the vessels extending in the blastema, BMP2 immunoreactivity was not observed in their cytoplasm. Slight reactions appeared to be distributed along their equator, where Newtic1 immunoreactivity (red) was seen, but

this could be due to the thick optical sections. In fact, analysis of blood cells collected from the blastema (Figure 7D) showed no BMP2 immunoreactivity at the margins of PcNobs. (**D-F**) A representative set of confocal images of TGFβ1 and Newtic1 double stain. TGFβ1 immunoreactivity (green), most of which was observed in PcNobs that had accumulated in blood vessels, was not detected in their cytoplasm and appeared to be distributed granularly on the equator where Newtic1 immunoreactivity (red) was seen. This pattern of immunoreactivity, unlike that of BMP2, was also observed in blood cells collected from the blastema (Figure 7E). (**G-I**) A representative set of confocal images of RFP and Newtic1 double stain for the control. Green: RFP; Red: Newtic1; Blue: TO-PRO-3 nuclear stain. Note that all primary antibodies used here were produced by a rabbit. Therefore, staining with Newtic1 antibody was preceded by staining with the other primary antibodies. Scale bars: 40 μm.

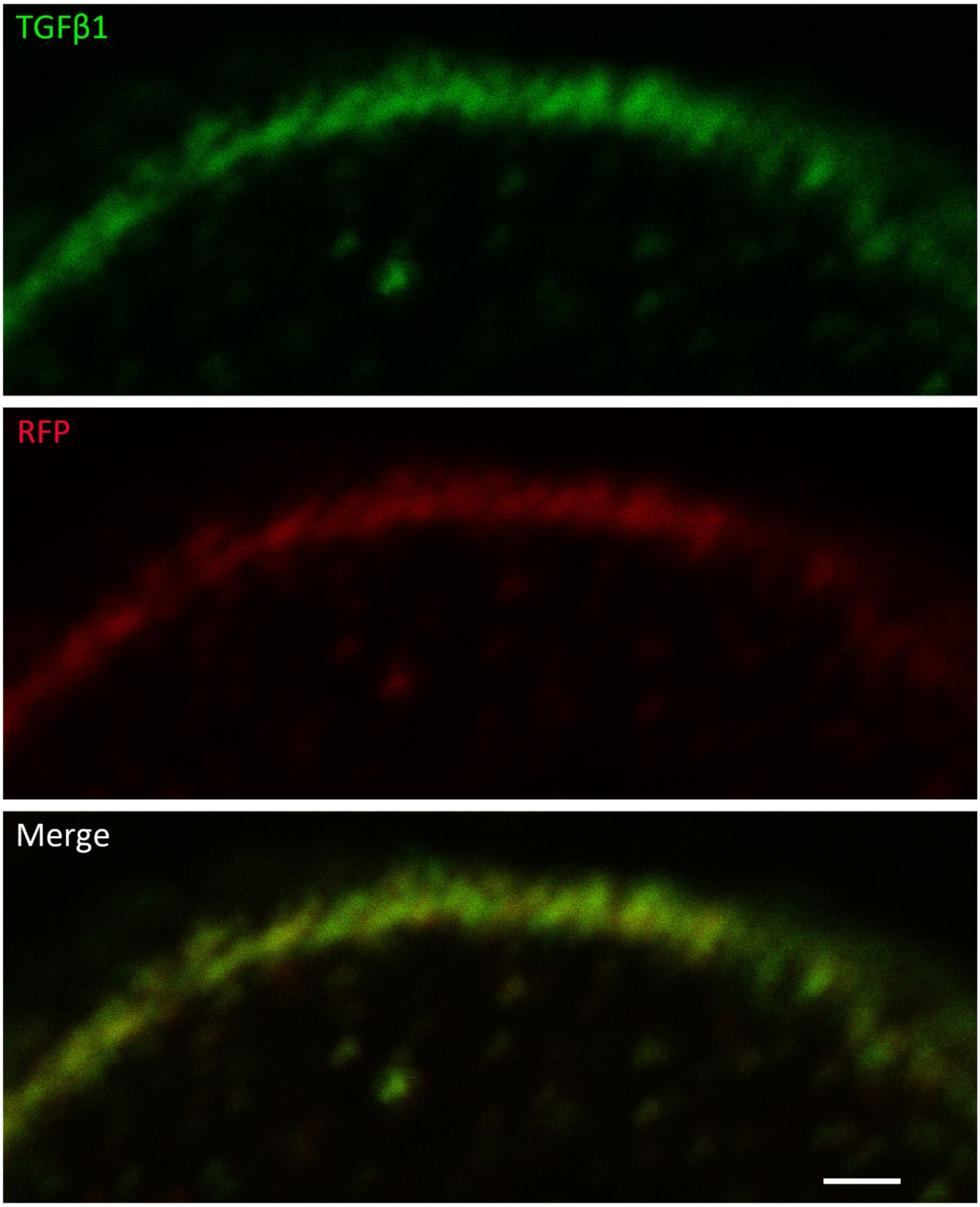

Supplementary Figure S4. A representative set of TGFβ1 and RFP double staining in PcNobs (n=4). Since primary antibodies used here were produced by a rabbit, staining with RFP antibody was preceded by staining with TGFβ1 antibody. Fluorescence observed in RFP is due to cross reaction of the secondary antibody for RFP to the primary antibody for TGFβ1. Scale bar: 1 µm.
